# Leveraging Large Language Models for Accurate AO Fracture Classification from CT Text Reports

**DOI:** 10.1007/s10278-025-01603-6

**Published:** 2025-07-07

**Authors:** Markus Mergen, Daniel Spitzl, Conrad Ketzer, Maximilian Strenzke, Alexander W. Marka, Marcus R. Makowski, Keno K. Bressem, Lisa C. Adams, Florian T. Gassert

**Affiliations:** 1 Department of Diagnostic and Interventional Radiology, School of Medicine, TUM University Hospital, Technical University of Munich, 81675 Munich, Germany; 2 Department of Trauma Surgery, School of Medicine, TUM University Hospital, Technical University of Munich, 81675 Munich, Germany; 3 Department of Cardiovascular Radiology and Nuclear Medicine, School of Medicine and Health, German Heart Center Munich, Technical University of Munich, 80636 Munich, Germany

**Keywords:** Large language models, Decision support, Computer-aided diagnosis, Fracture

## Abstract

Large language models (LLMs) have shown promising potential in analyzing complex textual data, including radiological reports. These models can assist clinicians, particularly those with limited experience, by integrating and presenting diagnostic criteria within radiological classifications. However, before clinical adoption, LLMs must be rigorously validated by medical professionals to ensure accuracy, especially in the context of advanced radiological classification systems. This study evaluates the performance of four LLMs—ChatGPT-4o, AmbossGPT, Claude 3.5 Sonnet, and Gemini 2.0 Flash—in classifying fractures based on the AO classification system using CT reports. A dataset of 292 fictitious physician-generated CT reports, representing 310 fractures, was used to assess the accuracy of each LLM in AO fracture classification retrospectively. Performance was evaluated by comparing the models’ classifications to ground truth labels, with accuracy rates analyzed across different fracture types and subtypes. ChatGPT-4o and AmbossGPT achieved the highest overall accuracy (74.6 and 74.3%, respectively), outperforming Claude 3.5 Sonnet (69.5%) and Gemini 2.0 Flash (62.7%). Statistically significant differences were observed in fracture type classification, particularly between ChatGPT-4o and Gemini 2.0 Flash (Δ12%, *p* < 0.001). While all models demonstrated strong bone recognition rates (90–99%), their accuracy in fracture subtype classification remained lower (71–77%), indicating limitations in nuanced diagnostic categorization. LLMs show potential in assisting radiologists with initial fracture classification, particularly in high-volume or resource-limited settings. However, their performance remains inconsistent for detailed subtype classification, highlighting the need for further refinement and validation before clinical integration in advanced diagnostic workflows.

## Introduction

Orthopedic trauma care relies on precise fracture classification to guide treatment and prognostication. The AO/OTA system, which categorizes fractures by bone, segment, and type, serves as the global standard for this purpose [1]. While effective, its complexity demands expertise, often leading to interobserver variability [2]. Artificial intelligence (AI) tools have emerged to address this challenge, with prior work focusing on image-based deep learning models achieving 69–88% accuracy in fracture detection [3]. However, the potential of large language models (LLMs) to interpret textual radiology reports for AO classification remains unexplored—a critical gap given the ubiquity of narrative-style CT/MRI reports in clinical workflows [4].

Recent advances in natural language processing (NLP), particularly LLMs like GPT-4o, have demonstrated promise in extracting structured insights from clinical text [5]. However, prior work has predominantly focused on Electronic Health Record (EHR) data or imaging (e.g., tumor measurements) rather than descriptive text [6–9]. CT reports pose unique NLP challenges: they combine standardized lexicon with free-text observations, contain ambiguous modifiers, and often omit explicit diagnostic conclusions [10].

Although convolutional neural networks (CNNs) have been widely utilized for lesion classification based on pixel-level image data [11], the capability of large language models (LLMs) to interpret and integrate textual information remains relatively underexplored [12, 13]. This represents a significant gap, as radiologic decision-making inherently combines visual patterns with contextual clinical information—an integration often reflected in radiology report narratives. Initial approaches to classifying these reports relied on rule-based systems [14] and traditional machine learning pipelines. While these methods showed some promise, they frequently struggled to capture the subtlety and complexity of the language commonly found in free-text clinical documentation. In contrast, large language models (LLMs) have the potential to directly infer diagnostic reasoning from unstructured text, offering adaptability to linguistic variation without the need for manual feature engineering. Early research on extracting TNM classifications from free-text radiology reports has demonstrated encouraging initial results [15].

General-purpose LLMs like ChatGPT-4o (OpenAI) and Gemini 2.0 Flash (Google) demonstrate remarkable text-processing capabilities but lack medical specialization [16]. In contrast, AmbossGPT—trained exclusively on the curated Amboss medical library—represents a domain-adapted alternative. We hypothesize that such clinical tailoring improves AO classification accuracy, particularly for nuanced fracture subtypes.

This study evaluates four LLMs (ChatGPT-4o May 2024 release, AmbossGPT, Claude 3.5 Sonnet, Gemini 2.0 Flash) on 292 fictitious physician-generated CT reports. These reports were meticulously designed to reflect the typical linguistic patterns, terminology, and structural nuances found in authentic clinical practice, ensuring external validity despite the artificial nature of the dataset. By benchmarking performance across anatomical regions and AO subcodes, we aim to (1) establish baseline accuracy for text-based LLM fracture classification, (2) identify error patterns limiting clinical utility, and (3) guide future development of a specialized diagnostic tool. To show generalizability of our findings, we conducted a real-life validation using the LLaMA 3.3–70B model using 141 radiology reports, achieving an overall accuracy of 69.9%.

## Methods

### Study Design and Population

Approval from an institutional review board for our fictitious cohort was not required due to the use of nonidentifiable data. This study analyzed 310 AO-classified fractures from 292 fictitious CT reports generated by two expert radiologists (two and three years of experience, respectively) and one expert trauma surgeon (two years of experience). The reports were systematically created to closely resemble real-world clinical radiology reports in terms of structure, terminology, and level of detail. To ensure consistency and standardization, a predefined template was used to guide the generation of reports, covering essential elements such as fracture description, localization, and classification. The template was designed based on established radiological reporting standards and adapted to reflect common linguistic patterns observed in real clinical documentation. Each CT report explicitly documented fractures. Fractures were limited to a maximum of two per report. The case distribution reflects a heterogeneous cohort, representative of the typical clinical spectrum encountered in trauma CT imaging. Each report was independently reviewed by at least one additional expert to ensure accuracy and adherence to the standardization criteria. Discrepancies in fracture descriptions or classifications were resolved through consensus discussions among the three medical experts. Ambiguous phrasing (e.g., “possible instability”) was retained to simulate real-world variability.

For the real-life validation cohort, a total of 145 fractures from 141 radiology reports were analyzed using the same prompting strategy as for the artificial reports. We employed the LLaMA 3.3–70B language model on a secure hospital server. The study was conducted in accordance with local ethical guidelines under ethics approval number 2024–590-S-CB.

### LLM Inference

Four models were evaluated in a zero-shot setting:

- ChatGPT-4o (May 2024 version, gpt-4o-2024–05-13).
- AmbossGPT (v2.1.0, Amboss medical library cutoff: April 2024).
- Claude 3.5 Sonnet (claude-3–5-sonnet-20240620).
- Gemini 2.0 Flash (gemini-2.0 flash experimental).
- For the real-world validation cohort, we used Llama 3.3–70b on a secure on-site server.

Prompts followed a standardized template:

“Provide me with only the AO classification according to the AO/OTA version of 2018 for the following findings, following the example schema 11A.”

### Statistical Analysis

The dataset size was chosen to ensure a balanced representation of different fracture patterns while maintaining practical feasibility. We performed a precision-based sample size estimation to justify the dataset size for evaluating classification performance. Assuming an expected overall classification accuracy of 70%—based on prior studies using deep learning for fracture detection—and aiming for a 95% confidence interval with a maximum width of ± 5%, the minimum required sample size was calculated as 323 fractures.

Accuracy was defined as the proportion of cases in which the exact AO code was correctly predicted against ground truth, including all three components: bone, segment, and fracture type. The AO classification system assigns fractures based on these elements, and a match was considered correct only if all components were accurately identified. Additionally, subclassification accuracy was calculated separately for each component—bone, segment, and fracture type—to evaluate performance on these individual levels. The Python packages pandas (version 2.2.0), matplotlib (version 3.8.2), and seaborn (version 0.13.2) were used for data analysis and visualization [17–19]. Performance differences were assessed using statistical tests to compare both overall classification accuracy and subclassification accuracy between groups or methods.

To account for multiple comparisons, we applied Holm-Bonferroni correction across all pairwise McNemar tests comparing LLM classification accuracy. This approach controls the family-wise error rate and ensures robust inference across six model comparisons. Adjusted *p*-values are reported alongside unadjusted values in the Results section.

For each accuracy estimate, we computed 95% confidence intervals using nonparametric bootstrapping (10,000 resamples with replacement), reporting the 2.5th and 97.5th percentiles of the bootstrap distribution as the lower and upper bounds.

## Results

**Fig. 1:**
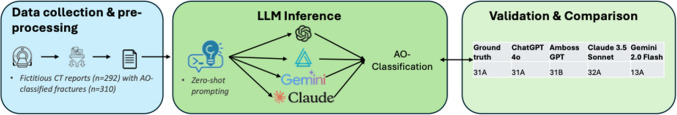
Schematic representation of the workflow for classifying fractures into the AO system based on CT reports. The process includes CT report extraction, LLM-based classification, and validation against ground truth

Fractures spanned 8 anatomical regions: femur (22.3%, *n* = 69), forearm (30.6%, *n* = 95), spine (7.7%, *n* = 24), pelvis (4.2%, *n* = 13), hand (4.2%, *n* = 13), humerus (17.7%, *n* = 55), lower leg (8.7%, *n* = 27), and other (e.g., scapula or clavicle; 4.5%, *n* = 14). Fractures were classified into proximal, diaphyseal, and distal regions of the bone where applicable. Since not all fractures could be categorized in this way, the numbers are lower than the total fracture count. Of the fractures that could be classified, 52.7% (*n* = 135) were located in the proximal region, 8.6% (*n* = 22) in the diaphyseal region, and 38.7% (*n* = 99) in the distal region. Fractures were classified into types A, B, and C according to the AO classification. Type A fractures accounted for 36.1% (*n* = 112), type B fractures for 29.4% (*n* = 91), and type C fractures for 34.5% (*n* = 107). Study workflow can be seen in Fig. 1.

### Overall Performance

**Fig. 2:**
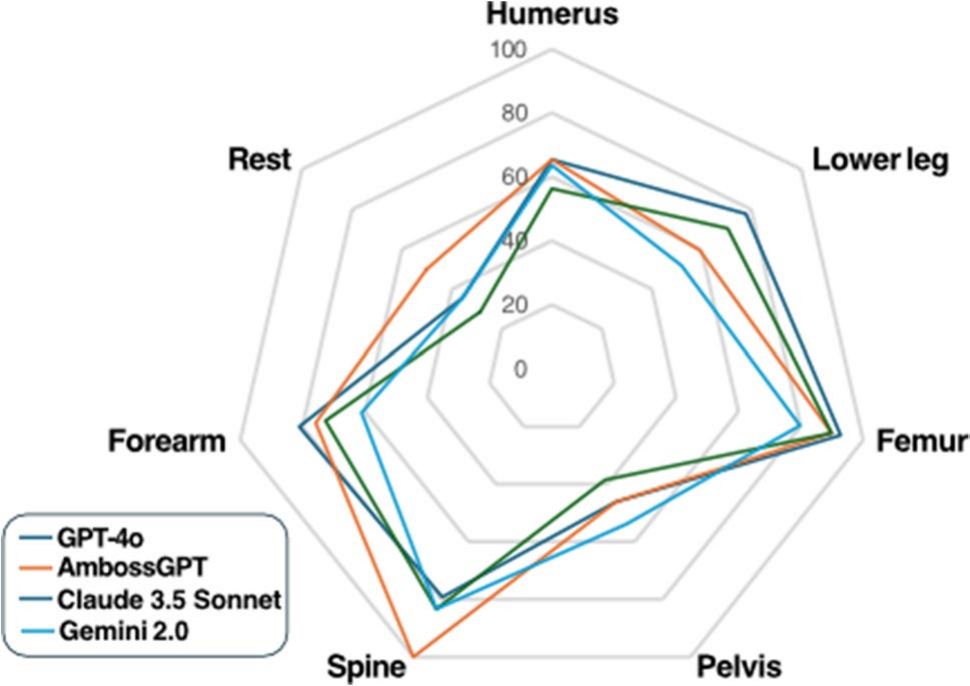
Radar chart illustrating the accuracy for correct AO classification by GPT-4o, AmbossGPT, Claude 3.5 Sonnet, and Gemini 2.0 Flash across anatomical regions. Key findings include AmbossGPT’s high accuracy (100%) for spinal fractures and ChatGPT-4o’s strong performance in femur fractures (92.9% accuracy)

**Fig. 3:**
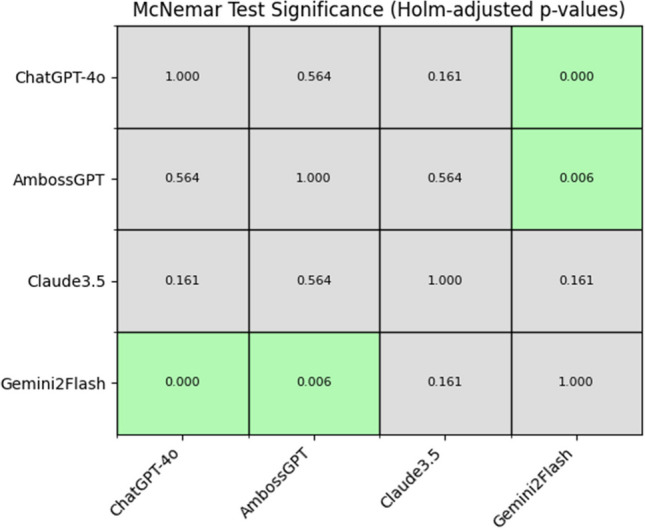
Statistical significance matrix based on pairwise comparisons using McNemar’s test using Holm-Bonferroni correction for multiple testing. ChatGPT-4o and AmbossGPT performed significantly better than Gemini (*p* < 0.05) in predicting accurate AO classification. No significant difference was observed between model performance for the other models used

ChatGPT-4o achieved the highest overall accuracy (Fig. 2) (74.6%), followed by AmbossGPT (74.3%), Claude 3.5 Sonnet (69.5%), and Gemini 2.0 Flash (62.7%). McNemar’s test: ChatGPT-4o and AmbossGPT performed significantly better than Gemini (*p* < 0.05) in predicting accurate AO classification. No significant difference was observed between model performance for the other models used (Fig. 3).

### Anatomical Region-Specific Performance

The models exhibited significant variation in accuracy across anatomical regions (Fig. 2). AmbossGPT achieved perfect accuracy (100%) for spinal fractures (*n* = 24/24), outperforming ChatGPT-4o (79.2%), Claude 3.5 Sonnet (83.3%), and Gemini 2.0 Flash (83.3%). Conversely, Gemini 2.0 Flash underperformed in hand fractures (7.7% accuracy, *n* = 1/13), misclassifying 12 of 13 cases, mostly misclassified the bone (11 of 13 cases). ChatGPT-4o demonstrated strong performance in femur fractures (92.9% accuracy, *n* = 65/70), while all models struggled with pelvic fractures (38.5–53.8% accuracy).

### Subclassification Accuracy

**Fig. 4:**
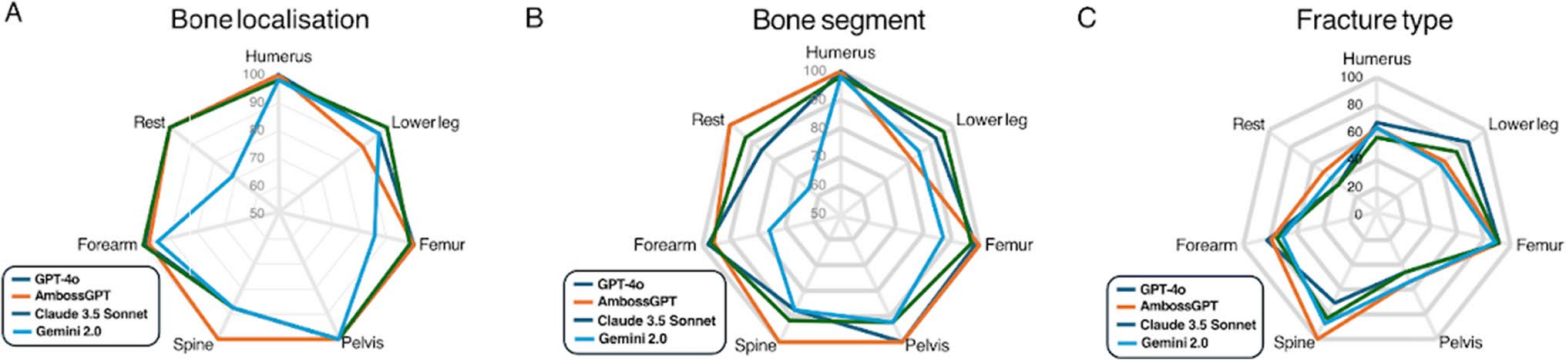
**A** Radar chart showing LLM accuracy in identifying the correct bone localization. All models performed well, with Claude 3.5 Sonnet achieving the highest accuracy (99.4%), followed by AmbossGPT (97.8%), ChatGPT-4o (96.1%), and Gemini 2.0 Flash (90.4%). **B** Radar chart illustrating LLM accuracy in localizing the correct bone segment. AmbossGPT led with 96.1% accuracy, closely followed by ChatGPT-4o (94.5%) and Claude 3.5 Sonnet (95.5%), while Gemini 2.0 Flash trailed at 81.4%. **C** Radar chart depicting LLM accuracy in classifying the correct fracture type. This task proved most challenging, with ChatGPT-4o (76.2%) and AmbossGPT (77.5%) outperforming Claude 3.5 Sonnet (72.7%) and Gemini 2.0 Flash (71.1%)

**Fig. 5:**
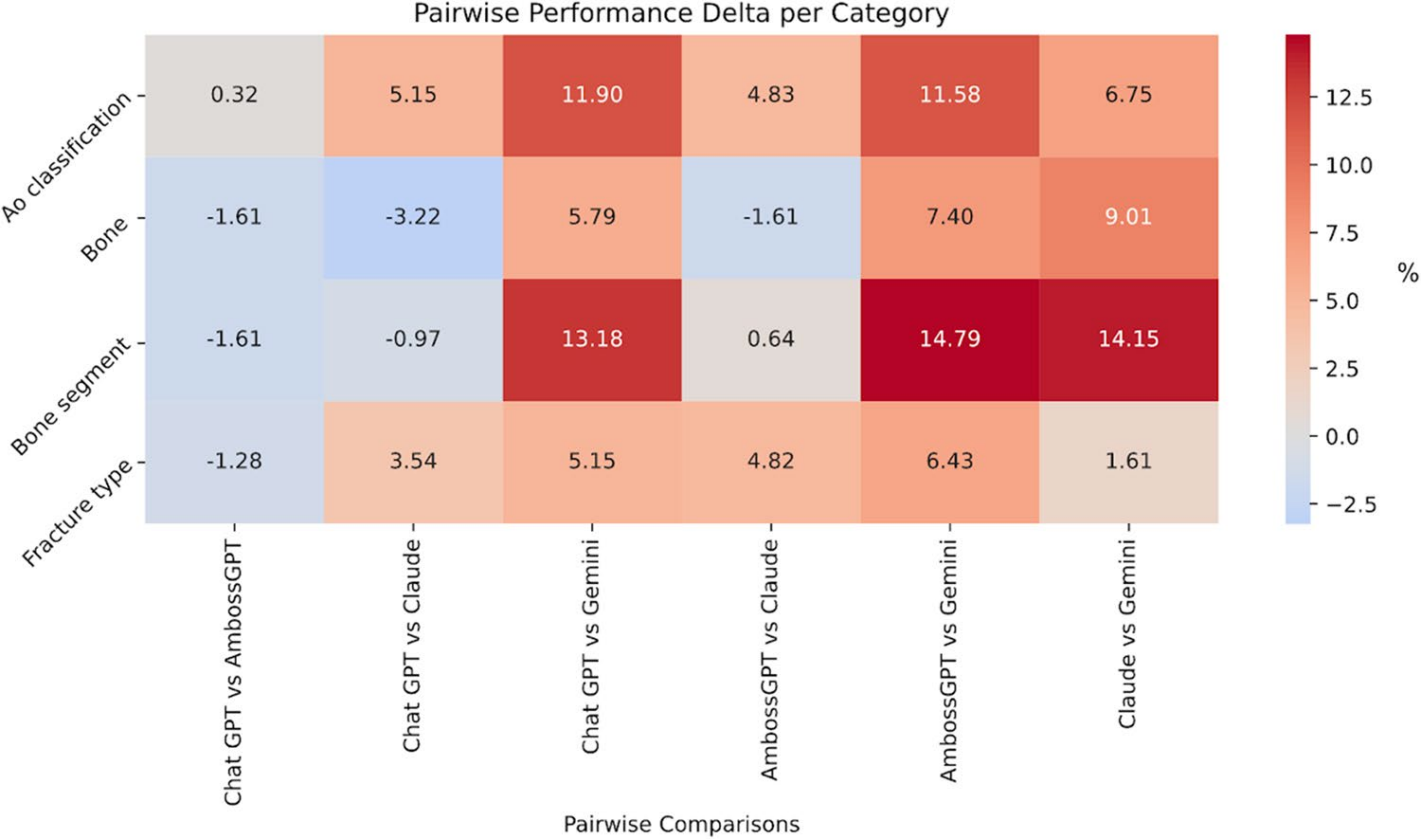
A heatmap showing pairwise model accuracy differences for overall AO, bone, bone segment, and fracture type classification

All models excelled in bone identification (90.4–99.4% accuracy; Fig. 4A) and segment localization (81.4–96.1%; Fig. 4B), but fracture type classification proved challenging (71.1–77.5% accuracy; Fig. 4C). Claude narrowly outperformed others in bone recognition (99.4%), while AmbossGPT led in segment accuracy (96.1%). The largest performance gap occurred in fracture type classification, where ChatGPT-4o (76.2%) and AmbossGPT (77.5%) surpassed Claude 3.5 Sonnet (72.7%) and Gemini 2.0 Flash (71.1%) by 4.8–6.4 percentage points (*p* < 0.01). Performance differences in predicting the correct AO, bone, bone segment, and fracture type classification can be seen in Fig. 5.

### Real Life Validation Cohort

In the validation phase, we applied the LLaMA 3.3–70B to two datasets, each comprising 141 radiology reports: one randomly selected part of our previously used fictitious and one real-world cohort. The model’s performance was assessed across four key categories: overall performance, bone recognition, bone part recognition, and fracture type recognition (Table 1).

**Table 1:** Performance comparison of LLaMA 3.3–70B on fictitious and real-world radiology reports across four categories: overall performance, bone recognition, bone part recognition, and fracture type recognition. Shown is accuracy

Cohort	Overall accuracy	Bone	Bone part	Type
Fictitious	0.697	0.945	0.917	0.738
Real world	0.699	0.986	0.938	0.726

For the fictitious dataset, the results were as follows: overall performance 69.7%, bone recognition 94.5%, bone part recognition 91.7%, and fracture type recognition 73.8%.

For the real-world dataset, the results were overall performance 69.9%, bone recognition 98.6%, bone part recognition 93.8%, and fracture type recognition 72.6%.

A McNemar test comparing the two datasets yielded a *p*-value of 0.784, indicating no statistically significant difference between the fictitious and real-world validation results.

## Discussion

This study provides the first comprehensive evaluation of LLMs for AO fracture classification using fictitious CT reports, revealing critical insights into their strengths and limitations. While AmbossGPT and ChatGPT-4o demonstrated promising accuracy (~ 74%), their performance lagged behind human experts (typically > 95% for AO coding [20]), particularly in fracture type classification—a gap that underscores the challenges of translating textual nuance into standardized codes. To demonstrate the generalizability of our findings, we performed a real-life validation using LLaMA 3.3–70B on 141 radiology reports, achieving an overall accuracy of 69.9%, closely mirroring the results obtained on the fictitious dataset.

The stark contrast between LLMs’ femur accuracy (80–93%) and hand failures (8–39%) mirrors known imbalances in fracture epidemiology and training data [21, 22]. Hand/pelvis fractures are less common, whereas femur fractures dominate trauma datasets, enabling better pattern recognition [23, 24]. This bias could be mitigated by synthetic data augmentation targeting rare fractures. While dataset biases likely contribute to these discrepancies, it is important to acknowledge that even human experts exhibit lower classification accuracy in complex regions such as the pelvis. Prior studies have shown that AO classification of pelvic fractures is subject to significant interobserver variability, particularly among non-specialists [25].

From a practical standpoint, LLMs could draft preliminary classifications for radiologist review, potentially reducing workloads [26]. However, their integration into clinical workflows requires human oversight, particularly for error-prone regions like the pelvis and hand [27]. Standardized reporting templates that minimize linguistic variability (e.g., replacing “minimally displaced” with AO-defined terms like “non-displaced”) could further enhance reliability [28]. However, Integration challenges, such as legal, ethical, and accountability issues, will have to be addressed.

Recent advances in medicine and radiology have sparked significant interest in the potential applications of large language models (LLMs) for interpreting medical data. For instance, LLMs have demonstrated the ability to provide accurate answers to medical questions related to lung cancer [29] and to generate clinically relevant impressions for radiology reports [30]. They have also been employed to convert free-text radiology reports into structured formats [31, 32], including in multilingual settings [33]. Furthermore, LLMs have shown promise in supporting clinical decision-making in oncology and facilitating the application of various clinical guidelines [34–39].

Similar studies have already demonstrated that LLMs can be effectively applied to various radiological classification tasks. For instance, one study [12] automatically extracted the LI-RADS score from radiology reports. In another study [15], LLMs were used to perform the TNM classification for NSCLC by identifying key tumor characteristics from free-text CT reports and converting them into standardized classifications. Additionally, LLMs have been successfully employed in the classification of brain tumors [40], where they distilled complex diagnostic information from radiology reports to support precise diagnostic decisions.

In contrast, there are currently no studies that specifically address the classification of fractures using LLMs based solely on CT reports. However, the findings of the present study emphasize the need to benchmark LLM performance against that of human experts with varying levels of domain expertise in a prospective setting, further validating our findings that LLMs could assist clinicians.

A primary limitation of this study is the use of fictitious, physician-generated CT reports from a single center. While this approach circumvents ethical and privacy barriers, it may not capture the full linguistic variation seen in multi-institutional or real clinical data. However, because these reports were created by board-certified radiologists and trauma surgeons specifically to mirror real-world phrasing and complexity, they provide a strong initial testbed for evaluating LLM accuracy. Future studies should validate these findings on large, multi-center datasets of authentic clinical reports. Additionally, our classification focus was limited to textual inputs without integrating imaging data; multimodal LLMs may substantially improve performance on challenging fracture subtypes. Integrating imaging data through multimodal architectures could address gaps in fracture type recognition, like proposed by Wang et al. by integrating LLMs with computer-aided diagnosis networks [35].

Future research should prioritize three directions. First, developing multimodal LLMs that fuse radiology text with imaging embeddings could bridge the gap between narrative descriptions and visual findings. Second, while zero-shot prompting allowed for efficient large-scale evaluation without extensive task-specific fine-tuning, it inherently constrained the model’s ability to fully capture clinical nuance. Future work incorporating few-shot learning or domain-adapted prompting may enhance both accuracy and contextual understanding. Fine-tuning models on AO classification guidelines via retrieval-augmented generation (RAG) may improve subtype accuracy.

Third, prospective studies are needed to assess how LLM-assisted workflows impact diagnostic speed, surgeon-radiologist communication, and patient outcomes in emergency settings.

Our findings suggest that LLMs, when carefully validated, could reliably assist in the initial classification of fractures based on textual CT reports. While human oversight remains crucial, these models have the potential to streamline radiological workflows, especially for common fracture types. Future work should expand these methods to multicenter and multimodal data, aiming to refine and validate their performance across a broader spectrum of clinical scenarios.

## Data Availability

The data that support the findings of this study are available from the corresponding author, [Daniel Spitzl], upon reasonable request.
